# Identification of a Novel Circular DNA Virus in New Zealand Fur Seal (*Arctocephalus forsteri*) Fecal Matter

**DOI:** 10.1128/genomeA.00558-13

**Published:** 2013-08-08

**Authors:** Alyssa Sikorski, Anisha Dayaram, Arvind Varsani

**Affiliations:** School of Biological Sciences, University of Canterbury, Christchurch, New Zealanda; Biomolecular Interaction Centre, University of Canterbury, Christchurch, New Zealandb; Electron Microscope Unit, Division of Medical Biochemistry, Department of Clinical Laboratory Sciences, University of Cape Town, Observatory, South Africac

## Abstract

Fur seal feces-associated circular DNA virus (FSfaCV) is a novel virus isolated from the fecal matter of New Zealand fur seals. FSfaCV has two main open reading frames in its 2,925-nucleotide (nt) genome. The replication-associated protein (Rep) of FSfaCV has similarity to Rep-like sequences in the *Giardia intestinalis* genome.

## GENOME ANNOUNCEMENT

A significant number of novel small circular DNA viruses have been identified and characterized from fecal sources over the past five years using viral metagenomic approaches (1–9). Most of these novel viruses are extremely diverse and have a variety of genomic architectures (10), and in most cases it has not been possible to assign these to the appropriate viral families. Nonetheless, these discoveries are helping to build data sets of small circular DNA viral genomes and thus will improve the overall resolution for classification and shed some light on their ecology.

A fecal sample from a New Zealand fur seal (*Arctocephalus forsteri*) was collected in October 2012 off the coast of Kaikoura, New Zealand. Approximately 5 g of fecal material was homogenized with 5 ml of SM buffer (0.1 M NaCl, 50 mM Tris-HCI [pH 7.4], 10 mM MgSO_4_). The slurry was centrifuged at 10,000 × *g* for 10 min and the supernatant was sequentially filtered through 0.45-µm and 0.2-µm syringe filters (Sartorius Stedim Biotech, Germany). The total viral DNA was isolated from 200 µl of the filtrate using the High Pure viral nucleic acid kit (Roche), and the circular viral DNA was enriched by rolling circle amplification (RCA) using the Illustra TempliPhi amplification kit (GE Healthcare), as described previously (8, 11–15). The enriched concatenated DNA was restricted with EcoRI endonuclease, yielding a 2.9-kb fragment that was purified and cloned into pGEM3ZF(+) (Promega) plasmid restricted with EcoRI and sequenced by primer walking.

BLASTn analysis of the 2,925-nucleotide (nt) sequence yielded no hits; however, we identified two putative major open reading frames (ORFs) (1,050 nt and 1,095 nt). The putative ORFs are bidirectionally arranged and separated by two intergenic regions (198 nt and 462 nt). The larger intergenic region contains a conserved nonanucleotide motif TAGTATTAC, which is similar to that found in most cycloviruses (16). A BLASTx analysis of the 1,050-nt ORF indicated that it potentially encodes a replication-associated protein (Rep) involved in the initiation of rolling circle replication. The putative Rep has similarities to the Rep-like sequences in the genomes of *Giardia intestinalis* (GenBank accession no. EES99726, 81% coverage, 29% identity, E value = 2 × 10^-20^), bat circovirus isolate XOR7 (GenBank accession no. KC339249, 80% coverage, 29% identity, E value = 1 × 10^-19^), and porcine circovirus 2 (GenBank accession no. JX945577, 82% coverage, 29% identity, E value = 2 × 10^-19^). Within the putative Rep, we identified the putative rolling circle replication motifs I, II, and III (LTVKN, HCHLNLEL, and YLAKDGEF, respectively) and the SF3 helicase Walker A and B motifs (GPAGSGKS and IWFDEFNG, respectively). No similarities were found for the 1,095-nt ORF by BLASTx analysis; however, we postulate that this ORF encodes the capsid protein. Additional analysis verified that we had in fact recovered, cloned, and sequenced the complete genome. We propose to name the novel isolate fur seal feces-associated circular DNA virus (FSfaCV) (GenBank accession no. KF246569).

Recently, Liu et al. (17) identified Rep-like integrons with similarities to single-stranded DNA (ssDNA) viruses in a variety of eukaryote genomes, including those of animals, plants, fungi, and protists; therefore, it is not surprising that the Rep of FSfaCV shares similarities with the Rep-like sequences found in the genome of *G. intestinalis.*

### Nucleotide sequence accession number.

The complete genome of FSfaCV has been deposited at GenBank under the accession no. KF246569.
